# Benchmarking publicly accessible large language models for high-myopia multiple-choice question generation in digital ophthalmic education and public health training

**DOI:** 10.3389/fpubh.2026.1843045

**Published:** 2026-05-05

**Authors:** Ligang Jiang, Xin Jiang, Wencan Wu, Fangzheng Jiang

**Affiliations:** 1 Department of Ophthalmology, Quzhou Affiliated Hospital of Wenzhou Medical University, Quzhou People’s Hospital, Quzhou, Zhejiang, China; 2 Quzhou College of Technology, Quzhou, Zhejiang, China; 3 The Eye Hospital, School of Ophthalmology & Optometry, Wenzhou Medical University, Wenzhou, China; 4 Oujiang Laboratory (Zhejiang Lab for Regenerative Medicine Vision and Brain Health), Wenzhou, Zhejiang, China; 5 Wenzhou Institute, University of Chinese Academy of Sciences, Wenzhou, China

**Keywords:** artificial intelligence, digital public health education, high myopia, large language models, multiple-choice questions, ophthalmology training

## Abstract

**Background:**

Digital tools are reshaping public health education and training, yet evidence on whether large language models (LLMs) can generate specialist ophthalmic teaching materials remains limited. High myopia (HM), a vision-threatening condition with long-term management needs and public health relevance, provides a suitable setting for evaluating this capability. This study compared five LLMs in generating HM-related multiple-choice questions (MCQs) for ophthalmic education.

**Methods:**

Five LLMs (ChatGPT-5.4, Gemini 3, DeepSeek, Kimi K2.5, and Doubao) completed 60 predefined HM MCQ generation tasks each, yielding 300 MCQs. A standardized blueprint covered four domains: basic knowledge, clinical cases, diagnosis and treatment decision-making, and screening/follow-up management. Objective evaluation included structural completeness, format compliance, keyed-answer accuracy, output features, and response time. Two ophthalmology experts rated six domains using 5-point Likert scales, and Spearman analyses examined associations among text features, response time, and expert ratings.

**Results:**

All models achieved 100.0% initial structural acceptability, structural completeness, and format compliance. Keyed-answer accuracy was highest for ChatGPT-5.4 and Gemini 3 (both 100.0%), followed by DeepSeek (98.3%) and Kimi K2.5 and Doubao (both 95.0%). Significant between-model differences were observed across all output features and response time (all *p* < 0.001). ChatGPT-5.4 generated the shortest stems, Gemini 3 the shortest explanations and fastest responses, and Kimi K2.5 and Doubao the longest explanations and total outputs. Inter-rater agreement was good (ICC range, 0.835–0.885). Significant differences were found in clarity, distractor quality, and mean subjective score (all *p* < 0.001), but not in content rigor, educational usefulness, cognitive-level alignment, or overall usability. DeepSeek achieved the highest median mean score, while direct usability was highest for ChatGPT-5.4 (91.7%) and Gemini 3 (90.0%). Content rigor was strongly associated with overall usability (*ρ* = 0.85, *p* < 0.05), whereas distractor quality was negatively associated with explanation length (*ρ* = −0.43, *p* < 0.05) and total output length (*ρ* = −0.37, p < 0.05).

**Conclusion:**

LLMs can reliably generate structurally valid HM-related MCQs under standardized Chinese prompting conditions. Their value may lie in supporting digital ophthalmic education and public health training, although expert oversight remains necessary because meaningful differences persist in factual accuracy, distractor quality, and direct usability.

## 1 Introduction

As digital technologies become increasingly integrated into medical education and healthcare systems, how to leverage innovative digital tools to improve the efficiency of professional training, optimize pathways for knowledge dissemination, and support health workforce capacity building has become an important issue in contemporary public health education (1–6). In the field of eye health, this issue is of particular practical relevance. The continuously rising prevalence of myopia has become a global public health challenge (7, 8), and high myopia (HM), as one of the highest-risk subtypes within the myopia spectrum and one associated with the greatest burden of complications (9), is closely linked not only to macular lesions, retinal detachment, choroidal neovascularization, glaucoma, and irreversible visual impairment (10), but also to increased demands for long-term screening, standardized referral, and ongoing follow-up management. Therefore, HM is no longer merely a refractive abnormality, but rather an important eye health issue characterized by a high disease burden, complex clinical management needs, and long-term implications for visual health (11, 12).

From an educational perspective, HM is likewise highly representative and comprehensive. Its related teaching content involves not only the mechanisms underlying the onset and progression of myopia, identification of risk factors, and interpretation of fundus and imaging features, but also the recognition and differential diagnosis of pathological myopia-related complications, as well as follow-up strategies, intervention decision-making, and patient education (13). This means that competency development related to HM is relevant not only to ophthalmology specialists, but also to primary eye care personnel, residents in standardized training programs, and other health professionals engaged in continuing professional development. For such disease-specific topics with complex knowledge structures and clear clinical application value, multiple-choice questions (MCQs) have long been among the most commonly used assessment tools in medical education because of their standardized structure, their ability to cover different levels of difficulty and cognition, and their suitability for large-scale teaching and assessment (14–16). However, the development of high-quality MCQs typically relies on repeated refinement by domain experts. It requires not only accurate and clear stems, parallel and well-constructed options, and plausible distractors, but also a single best answer and alignment with the pre-specified cognitive level. As a result, item bank development often faces substantial human resource demands and considerable quality-control pressure (17).

In recent years, large language models (LLMs) have developed rapidly and are emerging as important candidate tools in the digital transformation of medical education (18–21). Existing studies have shown that LLMs have been applied in multiple scenarios, including answering medical examination questions, case discussions, knowledge explanation, patient education, and educational content generation (22), demonstrating considerable potential in improving learning support efficiency and assisting instructional design. From the perspective of public health education and training, the potential value of such tools lies not only in “answering questions,” but also in their capacity to support the generation of standardized teaching materials, expand competency-oriented training resources, and improve educational accessibility in relatively resource-limited settings. At the same time, however, LLM outputs may still be affected by factual errors, unstable wording, opaque reasoning processes, and insufficient educational appropriateness (23). Therefore, before being adopted in formal teaching or assessment settings, they still require rigorous validation and expert oversight (24, 25).

In ophthalmology, although research on LLMs has increased rapidly in recent years, the available evidence has still largely focused on question-answering performance, examination-answering ability, or the quality of general ophthalmic consultation responses (26–28), whereas systematic research on disease-specific educational content generation remains limited. In particular, in the context of myopia and HM, existing studies have paid greater attention to patient question-answering, the quality of educational information, or the comprehensibility of myopia care recommendations (29), while giving insufficient attention to standardized item-generation ability for ophthalmic education and training. For HM, an eye health topic with high prevalence, long-term management needs, and substantial public health relevance, there is still insufficient evidence as to whether different LLMs can consistently generate MCQs that are accurate in content, standardized in structure, and of good educational value. This issue remains especially underexplored under conditions involving publicly accessible platforms, standardized Chinese prompts, and real-world user settings, where whether stable differences exist among models in terms of item generation quality, efficiency, and usability has not yet been adequately clarified.

Accordingly, this study aimed to use HM as a disease-specific scenario to construct a standardized blueprint for single-best-answer MCQ generation in ophthalmic education and to compare the performance of five publicly accessible LLMs in generating HM-related MCQs. Model-generated outputs were systematically evaluated across multiple dimensions, including structural completeness, format compliance, correct answer accuracy, textual output characteristics, generation efficiency, and expert subjective ratings. Through this study, we hope to provide empirical evidence, from the perspective of digitally empowered eye health education and training, for the development of HM-specific teaching resources, the optimization of human–AI collaborative item-writing workflows, and the appropriate application of digital educational tools in specialist medical training.

## 2 Materials and methods

### 2.1 Study design

**Figure 1:**
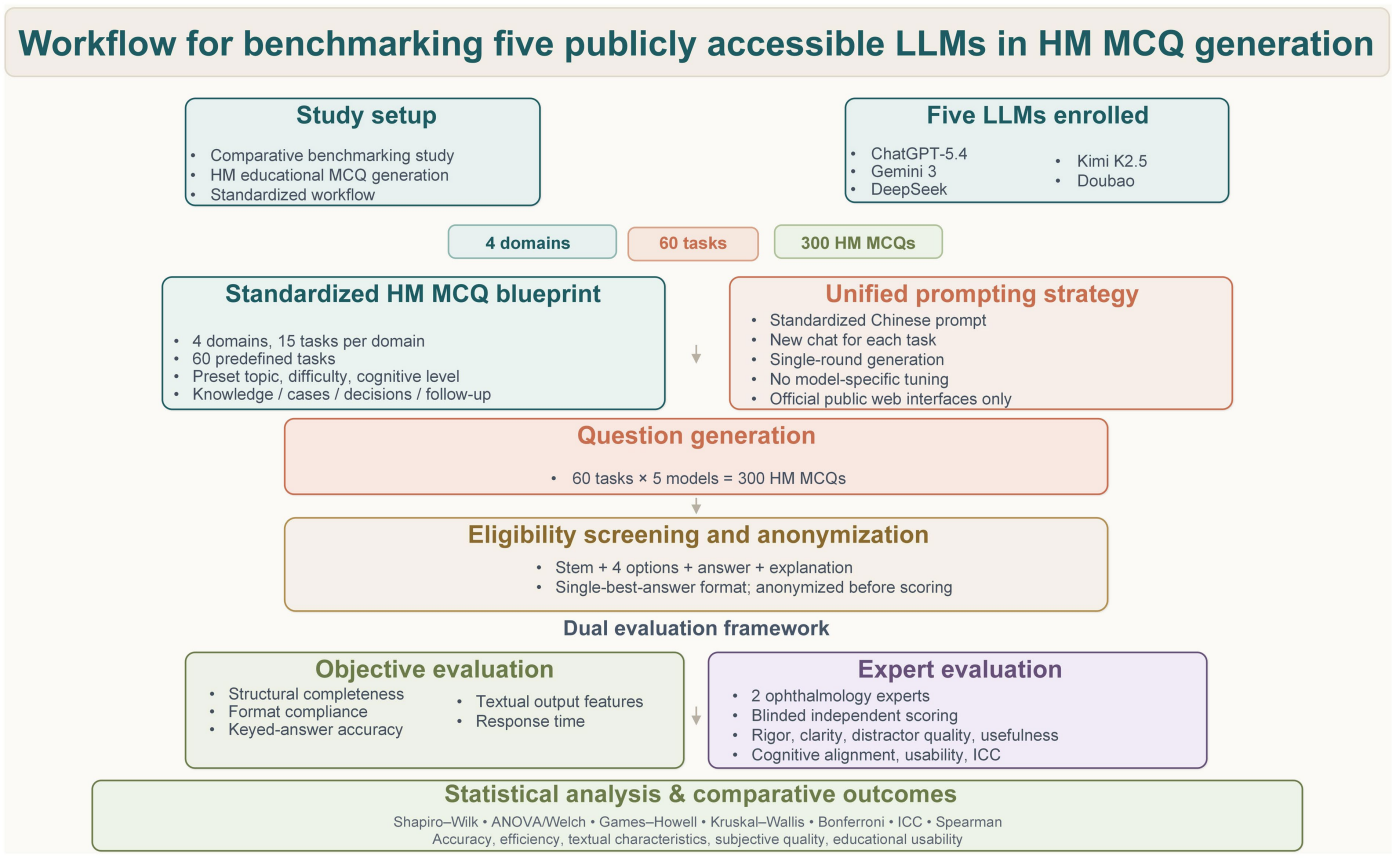
Study workflow for benchmarking five publicly accessible large language models (LLMs) in high myopia (HM) multiple-choice question (MCQ) generation. This summarizes the overall study design, including model enrollment, construction of the standardized HM MCQ blueprint, unified Chinese prompting strategy, question generation, eligibility screening and anonymization, dual evaluation framework, and final statistical analysis. A total of 60 predefined tasks across four domains were completed by each of the five models, yielding 300 HM-related MCQs for analysis.

This study was a comparative benchmarking study aimed at systematically evaluating performance differences among five publicly accessible LLMs in generating HM-related single-best-answer MCQs for ophthalmic education. The focus of the study was on the models’ item-generation capability and educational applicability, rather than their ability to answer pre-existing examination questions. To improve the fairness and comparability of cross-model comparisons, a unified HM item-generation blueprint was developed in advance, and model-generated outputs were systematically evaluated using a standardized Chinese prompt template, a standardized generation workflow, and a multidimensional evaluation framework (Figure 1).

### 2.2 Target models and access conditions

A total of five publicly accessible LLM chat systems were included in this study: ChatGPT-5.4, Gemini 3, DeepSeek, Kimi K2.5, and Doubao. All tests were conducted through the official public web interfaces of the respective platforms between March 10 and March 13, 2026. For each platform, priority was given to the high-performance thinking mode that could be directly identified and selected by ordinary users through the interface during the testing period.

Given the inconsistency in the degree to which underlying generation parameters are disclosed across platforms, this study did not attempt to forcibly standardize non-visible parameters across models. Instead, only user-controllable conditions were standardized, including access through the official web entry point, use of a uniform Chinese prompt template, completion of each task in a newly initiated independent conversation, and strict adherence to a consistent task-execution workflow. Any web search, code tools, or other enhanced functions that might have been available on a given platform were not actively enabled during formal testing. In addition, no model-specific prompt optimization was performed. Apart from one pre-specified standardized remedial prompt, no open-ended follow-up queries were added, and no multi-turn negotiated rewriting was used, so as to simulate a standardized horizontal comparison under real-world user conditions as closely as possible. All original dialog records and output content were fully preserved for subsequent data extraction, verification, and analysis.

### 2.3 Construction of the HM single-best-answer MCQ blueprint

Before formal testing, a standardized blueprint for single-best-answer MCQ generation was constructed around HM to unify the knowledge scope, competency levels, and structural requirements of the item-generation tasks across models. Based on the major teaching priorities and clinical training needs related to HM, the content was restricted to the HM educational scenario and divided into four pre-specified domains: (1) basic knowledge; (2) clinical cases; (3) diagnostic and therapeutic decision-making; and (4) screening and follow-up management.

Each content domain contained 15 task units, yielding a total of 60 standardized item-generation tasks. For each task unit, the content category, specific knowledge point, difficulty level, and cognitive level were pre-specified. Difficulty was categorized as easy, moderate, or difficult, and cognitive level was categorized as recall, comprehension, application, or analysis. Because mature methods for sample size calculation are currently lacking for this type of study, a pragmatic design was adopted to balance content coverage, representation across domains, and the feasibility of blinded expert rating. Ultimately, each model completed 60 pre-specified tasks, generating a total of 300 HM-related MCQs across the five models. The core purpose of this blueprint was to ensure that all models generated items for exactly the same educational tasks, thereby reducing bias arising from unequal knowledge-point distribution or differences in item structure.

### 2.4 Prompt design and item-generation workflow

A uniform Chinese prompt template was designed and used for all target models. The prompt required the model to generate one single-best-answer MCQ on a specified HM topic for ophthalmology residency training or related specialty teaching scenarios, and explicitly defined the topic, content category, specific knowledge point, difficulty level, cognitive level, output format, and item-writing constraints (Supplementary Appendix S1).

The standardized prompt required each item to include a stem, four options (A–D), one uniquely correct answer, and a brief explanation. The item was also required to conform to the single-best-answer MCQ format, with only one best answer permitted. Incorrect options were required to have a certain degree of distractive plausibility and educational value, but obviously unreasonable options, options semantically duplicative of the correct answer, and equivalent options that could also be considered correct in common clinical scenarios were not allowed. The stem was required to be clearly worded, logically coherent, and sufficiently informative, while avoiding double negatives, excessive cueing, or dependence on real images; if examination findings were involved, they could only be presented in textual form. The 60 pre-specified tasks were entered into each of the five models separately, with only one item generated per interaction, yielding a total of 300 model-generated items. In principle, all items were generated in a single round and completed sequentially according to the predefined task order. Only when the initial output failed to meet the pre-specified format requirements was one standardized remedial prompt allowed according to the predefined workflow for regeneration (Supplementary Appendix S1). If the regenerated output still failed to meet the requirements, it was recorded as a generation failure, retained for format analysis, but excluded from subsequent content-quality scoring.

### 2.5 Inclusion criteria and output processing

Model outputs were included in subsequent subjective quality scoring and primary analyses only when all of the following criteria were met simultaneously: (1) a complete stem; (2) four clearly labeled options from A to D; (3) an explicitly identified correct answer; (4) an explanation; (5) conformity with the single-best-answer MCQ format; and (6) only one defensible best answer. Outputs were judged structurally ineligible if any required field was missing, if multiple answers could reasonably be considered correct, if prohibited options such as “All of the above” or “None of the above” were used, or if the item could only be answered by relying on a real image that was not provided.

All items meeting the structural inclusion criteria were anonymized and presented in random order before expert rating to minimize rater bias as much as possible. Before formal evaluation, the original model outputs were not manually rewritten, factually corrected, or linguistically polished; except for necessary formatting adjustments, their original output characteristics were preserved as much as possible. Structurally ineligible outputs were excluded from subjective quality scoring, but were retained for analyses of generation failure rates and related objective indicators. In the present study, all 300 items generated by the five models met the structural inclusion criteria and entered subjective evaluation.

### 2.6 Reference key and evaluation manual

Before formal evaluation, the research team established a standardized reference key in advance on the basis of mainstream ophthalmology textbooks, HM- and pathological myopia-related guidelines, expert consensuses, and standardized training materials (30–34). This reference key was used to judge the accuracy of item content, whether the indicated correct answer was truly correct, and whether the explanation was consistent with the standard knowledge framework. The reference key covered the core content related to HM, including but not limited to definitions and classification, risk factors and pathogenesis, fundus and imaging features, pathological myopia-related complications, screening and follow-up strategies, and principles of intervention and management.

At the same time, the research team developed a standardized evaluation manual that pre-specified the objective evaluation indicators, Likert rating domains, judgment criteria, recording procedures, and discrepancy-resolution process, in order to improve the consistency of data extraction, subjective rating, and subsequent coding.

### 2.7 Objective evaluation indicators

A pre-specified objective evaluation framework was used to systematically assess the items generated by each model, mainly including the following aspects:

(1) Structural completeness: whether the model output contained all required components, including a stem, four options, the correct answer, and an explanation; the structurally complete output rate was then calculated.
(2) Format compliance: whether the model output conformed to the pre-specified single-best-answer MCQ format, including provision of four A–D options, explicit identification of one correct answer, and avoidance of multiple-answer structures and prohibited option formats.
(3) Correct answer accuracy: whether the answer indicated by the model was truly correct. Judgments were made against the pre-specified reference key. If the indicated answer was incorrect, ambiguous, inconsistent with the explanation, or lacked a reasonable medical basis, it was judged inaccurate.
(4) Textual output characteristics: given that Chinese word-segmentation methods may affect the stability of length statistics, character count was used as the primary text-length metric in this study. The recorded indicators included stem length, explanation length, total response length, mean option length, and option length SD. Option length SD was defined as the standard deviation of the character counts of the four answer options and was used to reflect whether option lengths were obviously imbalanced.
(5) Generation efficiency: operationalized as user-perceived latency under public web-based conditions rather than intrinsic model inference speed. Timing started when the prompt was submitted and ended when the model completed its visible output in the chat interface. Accordingly, this measure may reflect a combination of model behavior, server load, network latency, and time-dependent platform traffic.

### 2.8 Expert subjective ratings

Subjective quality evaluation was conducted independently by two ophthalmology experts, both of whom had more than 6 years of experience in ophthalmic clinical practice and medical education. Before formal rating, a small number of trial items were used for calibration to unify the rating scale and judgment standards. All included items were evaluated under anonymized and blinded conditions.

Each item was rated on a 5-point Likert scale across the following six domains:

(1) Content rigor: whether the item content, option design, and explanation were consistent with the mainstream ophthalmic educational knowledge framework and HM-related clinical knowledge;
(2) Clarity: whether the wording of the stem and options was clear, whether the logic was coherent, and whether the item was easy to understand;
(3) Distractor quality: whether the incorrect options had reasonable distractive plausibility and educational value;
(4) Educational usefulness: whether the item was suitable for ophthalmic teaching or formative assessment;
(5) Cognitive-level alignment: whether the generated item was consistent with the pre-specified cognitive level in the item-generation blueprint;
(6) Overall usability: whether the item could be used directly, could be used after minor revision, or could be used only after major revision.

In addition, to facilitate result presentation and applied interpretation, overall usability was further analyzed using a pre-specified three-category classification: items with an overall usability score of 5 were classified as “directly usable,” those with a score of 4 as “usable after minor revision,” and those with scores of 1–3 as “usable after major revision.”

To comprehensively reflect the overall subjective performance of each item, the arithmetic mean of the six subjective rating domains was further calculated, namely, the mean of the scores for content rigor, clarity, distractor quality, educational usefulness, cognitive-level alignment, and overall usability for each item. This metric was used for subsequent between-group comparisons and correlation analyses. It served only as a supplementary composite descriptive indicator for overall comparison and exploratory analysis, and did not replace the results of the individual rating domains.

### 2.9 Inter-rater agreement and discrepancy resolution

To assess the consistency of subjective ratings between the two experts, inter-rater agreement indices were calculated. For Likert-scale ratings, ICCs were used for evaluation, and 95% CIs were reported. According to the statistical settings of this study, ICCs were calculated using a single-measurement, two-way mixed-effects, consistency model. For key items such as correct answer judgment, structural eligibility, and overall usability classification, if there was a clear disagreement between the two experts, consensus was first reached through discussion; if necessary, a third senior ophthalmology expert was invited to arbitrate in order to obtain the final judgment. The judgment of overall usability classification was based on the pre-specified three-category rules.

### 2.10 Statistical analysis

Statistical analyses were performed using IBM SPSS Statistics 27.0. Categorical variables were presented as counts and percentages, whereas continuous variables were presented as mean ± SD or median (Q1, Q3) using a consistent descriptive format for each indicator across all models. For variables such as textual output characteristics and subjective ratings, normality was first assessed within each model using the Shapiro–Wilk test; if an indicator deviated from normality in any model, it was presented as median (Q1, Q3) for all models. In overall between-group comparisons, one-way ANOVA was used for continuous variables that were normally distributed and satisfied homogeneity of variances; when approximate normality was met but homogeneity of variances was not satisfied, Welch’s ANOVA was used, followed by Games-Howell *post hoc* pairwise comparisons. The Kruskal–Wallis test was used for non-normally distributed continuous variables or ordinal categorical data; when the overall difference was statistically significant, Bonferroni-adjusted pairwise comparisons were further performed, and the corresponding test statistics and adjusted *p*-values were reported. Agreement in expert subjective ratings was evaluated using ICCs, with 95% CIs reported. In addition, Spearman’s rank correlation analysis was used to assess the associations of textual output characteristics and response time with expert subjective ratings. All tests were two-sided, and *p* < 0.05 was considered statistically significant.

## 3 Results

### 3.1 Overall generation results and objective evaluation results

All five LLMs completed the 60 pre-specified HM single-best-answer MCQ generation tasks, yielding a total of 300 items. The initial outputs of all models met the structural inclusion criteria, and all final outputs satisfied the requirements for structural completeness and format compliance; therefore, all items entered subsequent subjective evaluation. During formal testing, no model triggered the standardized remedial prompt, and no generation failures occurred. As shown in Table 1, all five models achieved rates of 100.0% for structurally qualified initial outputs, final structurally complete outputs, format compliance, and eligibility for subjective evaluation. Accordingly, the objective differences among models were mainly reflected in correct answer accuracy. Specifically, correct answer accuracy was 100.0% for both ChatGPT-5.4 and Gemini 3, 98.3% for DeepSeek, and 95.0% for both Kimi K2.5 and Doubao.

**Table 1:** Objective evaluation results for HM educational MCQs generated by five LLMs.

Indicator	ChatGPT-5.4 (*n* = 60)	Gemini 3 (*n* = 60)	DeepSeek (*n* = 60)	Kimi K2.5 (*n* = 60)	Doubao (*n* = 60)
Structurally qualified on first output, *n* (%)	60 (100%)	60 (100%)	60 (100%)	60 (100%)	60 (100%)
Qualified after one standardized remedial prompt, *n* (%)	0 (0.0%)	0 (0.0%)	0 (0.0%)	0 (0.0%)	0 (0.0%)
Final structurally complete output, *n* (%)	60 (100%)	60 (100%)	60 (100%)	60 (100%)	60 (100%)
Format compliant, *n* (%)	60 (100%)	60 (100%)	60 (100%)	60 (100%)	60 (100%)
Correct answer accuracy, *n* (%)	60 (100%)	60 (100%)	59 (98.3%)	57 (95.0%)	57 (95.0%)
Eligible for subjective evaluation, *n* (%)	60 (100%)	60 (100%)	60 (100%)	60 (100%)	60 (100%)
Generation failure, *n* (%)	0%	0%	0%	0%	0%

Data are presented as *n* (%), with percentages calculated using 60 prespecified generation tasks per model as the denominator. “Structurally qualified on first output” indicates that the initial response met all prespecified structural inclusion criteria. “Qualified after one standardized remedial prompt” indicates outputs that became structurally eligible after one rescue prompt. “Final structurally complete output” indicates that the output contained a question stem, four A–D options, an explicitly identified correct answer, and an explanation. “Format compliant” refers to conformity with the prespecified single-best-answer multiple-choice question format. “Correct answer accuracy” was judged against the pre-established reference key. “Eligible for subjective evaluation” refers to structurally eligible outputs that entered blinded expert scoring. “Generation failure” refers to outputs that remained structurally non-compliant after the allowed remedial step. HM, high myopia; LLM, large language model; MCQ, multiple-choice question.

### 3.2 Textual output characteristics and generation efficiency

All five models showed statistically significant differences in stem length, mean option length, explanation length, total response length, option length SD, and response time (all *p* < 0.001; Table 2).

**Table 2:** Comparison of textual output characteristics and generation efficiency across five LLMs.

Indicator	ChatGPT-5.4 (*n* = 60)	Gemini 3 (*n* = 60)	DeepSeek (*n* = 60)	Kimi K2.5 (*n* = 60)	Doubao (*n* = 60)	Test statistic	*p*-values
Stem length	27.50 (25.00, 32.75)	35.50 (27.00, 45.00)	48.00 (30.00, 66.00)	49.00 (30.25, 57.50)	41.50 (26.25, 52.75)	46.604	**<0.001**
Mean option length	13.50 (10.81, 16.25)	10.88 (9.06, 14.13)	14.00 (9.56, 20.00)	16.00 (12.31, 19.19)	18.50 (12.50, 21.19)	41.232	**<0.001**
Explanation length	58.00 (54.00, 60.75)	39.00 (34.00, 45.00)	76.00 (71.00, 84.00)	110.00 (101.25, 117.75)	115.50 (98.00, 128.75)	255.093	**<0.001**
Total response length	141.00 (131.25, 153.75)	119.00 (111.00, 136.75)	186.50 (164.50, 208.75)	224.00 (206.50, 242.75)	224.00 (206.50, 247.75)	202.093	**<0.001**
Option length SD	4.33 (2.52, 5.75)	5.24 (2.59, 7.08)	6.09 (3.58, 10.06)	6.30 (3.99, 9.28)	4.54 (2.69, 5.98)	22.207	**<0.001**
Response time	10.36 ± 1.21	7.90 ± 0.80	17.71 ± 2.81	17.21 ± 3.01	12.22 ± 1.81	320.809	**<0.001**

Text-length variables are expressed as character counts. Option length SD refers to the standard deviation of the lengths of the four answer options. Response times are expressed in seconds. Stem length, mean option length, explanation length, total response length, and option length SD are presented as median (Q1, Q3). Response time is presented as mean ± SD. Overall between-model comparisons for stem length, mean option length, explanation length, total response length, and option length SD were performed using the Kruskal-Wallis test. Response time was analyzed using Welch’s ANOVA because homogeneity of variances was not satisfied. When the Kruskal-Wallis test was significant, Bonferroni-adjusted pairwise comparisons were performed. Pairwise comparisons for response time were performed using the Games-Howell test. Bold *p*-values indicate statistical significance (*p* < 0.05). LLM, large language model; Q1, first quartile; Q3, third quartile.

**Figure 2:**
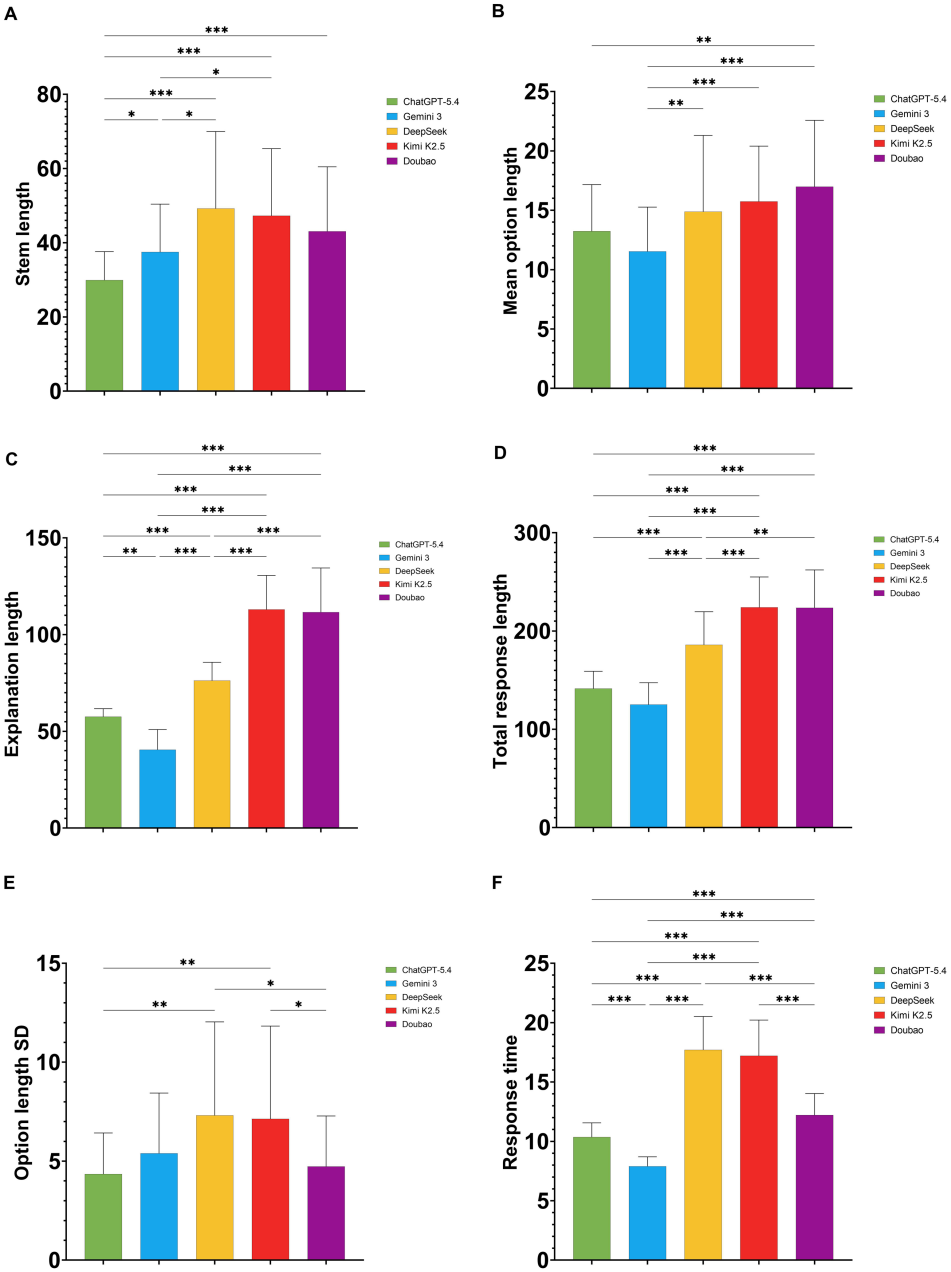
Comparison of textual output characteristics and generation efficiency across the five large language models (LLMs). **(A)** Stem length. **(B)** Mean option length. **(C)** Explanation length. **(D)** Total response length. **(E)** Option length standard deviation (SD). **(F)** Response time. Pairwise significant differences are indicated above the plots. *p < 0.05, **p < 0.01, ***p < 0.001.

For stem length, ChatGPT-5.4 generated the shortest stems, with a median of 27.50 (25.00, 32.75) characters. Gemini 3 generated stems of 35.50 (27.00, 45.00) characters, whereas DeepSeek and Kimi K2.5 produced relatively longer stems, at 48.00 (30.00, 66.00) and 49.00 (30.25, 57.50) characters, respectively. Doubao generated stems of 41.50 (26.25, 52.75) characters. *Post hoc* pairwise comparisons showed that ChatGPT-5.4 generated significantly shorter stems than the other four models, and Gemini 3 also generated significantly shorter stems than DeepSeek and Kimi K2.5 (Figure 2A).

For mean option length, Doubao generated the longest options, with a median of 18.50 (12.50, 21.19) characters, followed by Kimi K2.5 at 16.00 (12.31, 19.19) characters and DeepSeek at 14.00 (9.56, 20.00) characters. ChatGPT-5.4 generated options of 13.50 (10.81, 16.25) characters, whereas Gemini 3 generated the shortest options, at 10.88 (9.06, 14.13) characters (Figure 2B).

For explanation length, Kimi K2.5 and Doubao generated the longest explanations, with medians of 110.00 (101.25, 117.75) and 115.50 (98.00, 128.75) characters, respectively. DeepSeek ranked next, at 76.00 (71.00, 84.00) characters. By contrast, ChatGPT-5.4 and Gemini 3 generated relatively shorter explanations, with Gemini 3 being the shortest at 39.00 (34.00, 45.00) characters (Figure 2C).

The pattern for total response length was broadly consistent with that for explanation length. Kimi K2.5 and Doubao generated the longest total responses, both with a median of 224.00 characters, with interquartile ranges of 206.50 to 242.75 and 206.50 to 247.75, respectively. DeepSeek ranked next at 186.50 (164.50, 208.75) characters, whereas ChatGPT-5.4 and Gemini 3 generated shorter total responses, with Gemini 3 being the shortest at 119.00 (111.00, 136.75) characters (Figure 2D).

For option length SD, Kimi K2.5 and DeepSeek showed relatively higher values, at 6.30 (3.99, 9.28) and 6.09 (3.58, 10.06), respectively, indicating greater variability in option length across the four answer choices. Gemini 3 showed an intermediate value of 5.24 (2.59, 7.08), whereas ChatGPT-5.4 and Doubao showed relatively lower values, at 4.33 (2.52, 5.75) and 4.54 (2.69, 5.98), respectively (Figure 2E).

In terms of generation efficiency, Gemini 3 was the fastest, with a mean response time of 7.90 ± 0.80 s, followed by ChatGPT-5.4 at 10.36 ± 1.21 s and Doubao at 12.22 ± 1.81 s. DeepSeek and Kimi K2.5 required longer response times, at 17.71 ± 2.81 s and 17.21 ± 3.01 s, respectively. *Post hoc* comparisons showed that, except for the difference between DeepSeek and Kimi K2.5, which was not statistically significant, all other pairwise differences in response time were statistically significant (Figure 2F).

### 3.3 Expert subjective ratings and inter-rater agreement

**Figure 3:**
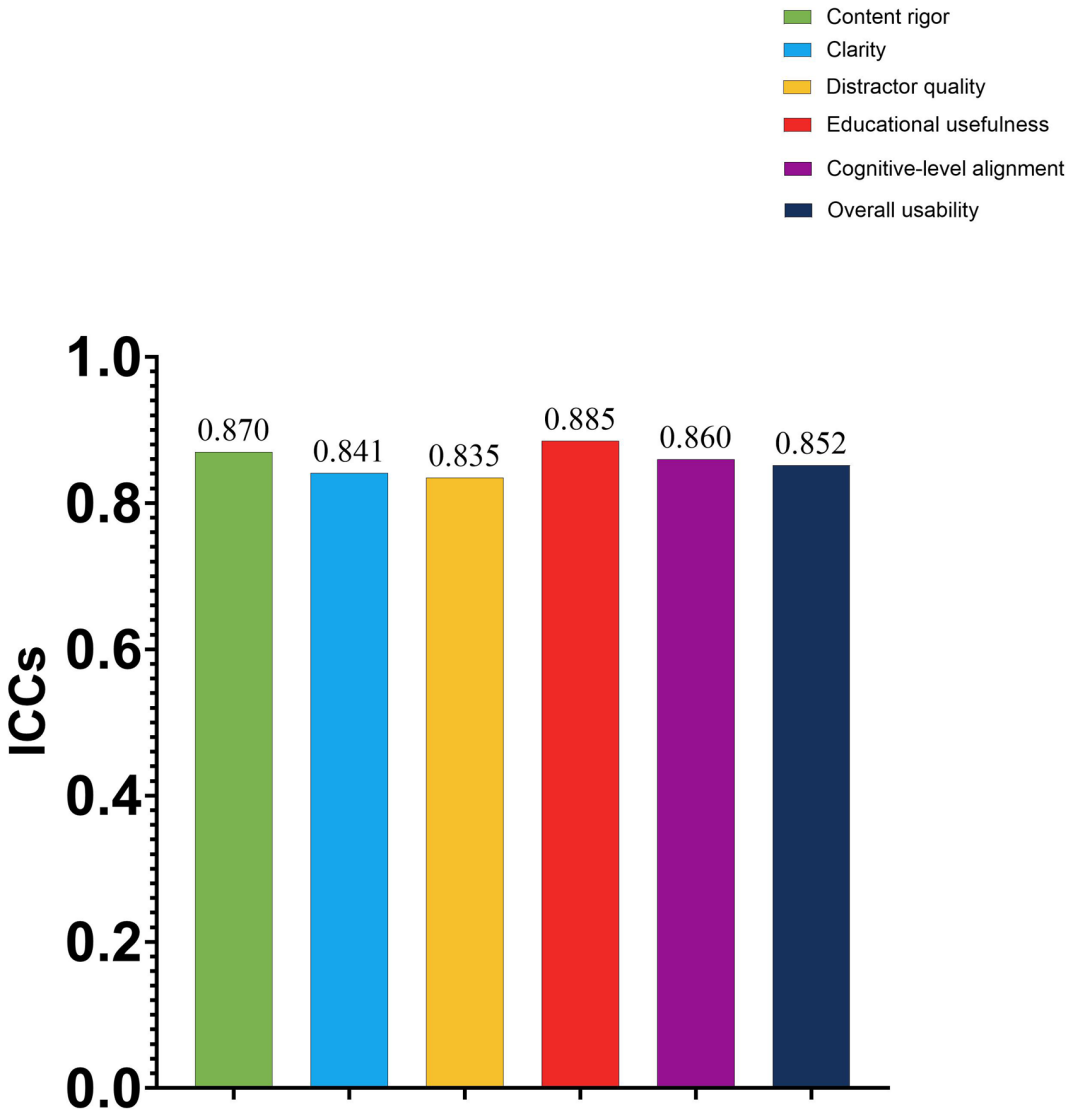
Inter-rater agreement for expert subjective ratings. Bar plots show the intraclass correlation coefficients (ICCs) for the six expert rating domains: content rigor, clarity, distractor quality, educational usefulness, cognitive-level alignment, and overall usability. All ICC values indicated good inter-rater agreement between the two ophthalmology experts.

All subjective rating outcomes were non-normally distributed and were therefore presented as median (Q1, Q3). Overall agreement between the two experts across all rating domains was good, with ICCs ranging from 0.835 to 0.885. Specifically, the ICC was 0.870 (95% CI: 0.839–0.895) for content rigor, 0.841 (95% CI: 0.804–0.871) for clarity, 0.835 (95% CI: 0.797–0.866) for distractor quality, 0.885 (95% CI: 0.858–0.908) for educational usefulness, 0.860 (95% CI: 0.827–0.887) for cognitive-level alignment, and 0.852 (95% CI: 0.818–0.881) for overall usability (Figure 3; Table 3).

**Table 3:** Comparison of expert subjective ratings across five LLMs.

Rating domain	ChatGPT-5.4 (*n* = 60)	Gemini 3 (*n* = 60)	DeepSeek (*n* = 60)	Kimi K2.5 (*n* = 60)	Doubao (*n* = 60)	ICC (95% CI)	*H*-values	*p*-values
Content rigor	5.00 (5.00, 5.00)	5.00 (5.00, 5.00)	5.00 (5.00, 5.00)	5.00 (5.00, 5.00)	5.00 (5.00, 5.00)	0.870 (0.839, 0.895)	6.507	0.164
Clarity	5.00 (5.00, 5.00)	5.00 (5.00, 5.00)	5.00 (5.00, 5.00)	5.00 (5.00, 5.00)	5.00 (4.00, 5.00)	0.841 (0.804, 0.871)	48.512	**<0.001**
Distractor quality	5.00 (5.00, 5.00)	5.00 (5.00, 5.00)	5.00 (5.00, 5.00)	4.00 (4.00, 4.00)	4.50 (4.00, 5.00)	0.835 (0.797, 0.866)	105.872	**<0.001**
Educational usefulness	5.00 (5.00, 5.00)	5.00 (4.63, 5.00)	5.00 (5.00, 5.00)	5.00 (5.00, 5.00)	5.00 (4.50, 5.00)	0.885 (0.858, 0.908)	5.863	0.210
Cognitive-level alignment	5.00 (5.00, 5.00)	5.00 (5.00, 5.00)	5.00 (5.00, 5.00)	5.00 (5.00, 5.00)	5.00 (5.00, 5.00)	0.860 (0.827, 0.887)	2.250	0.690
Overall usability	5.00 (5.00, 5.00)	5.00 (5.00, 5.00)	5.00 (5.00, 5.00)	5.00 (5.00, 5.00)	5.00 (4.13, 5.00)	0.852 (0.818, 0.881)	8.966	0.062
Mean score	4.92 (4.83, 5.00)	4.92 (4.83, 5.00)	5.00 (5.00, 5.00)	4.83 (4.75, 4.83)	4.83 (4.60, 4.92)	NA	56.019	**<0.001**

Data are presented as median (Q1, Q3) because all rating outcomes were non-normally distributed according to the Shapiro–Wilk test within each model. All rating domains were evaluated using a 5-point Likert scale. ICC values are single-measure intraclass correlation coefficients (two-way mixed-effects model, consistency type) with 95% confidence intervals. Overall between-model comparisons were performed using the Kruskal–Wallis test. Post hoc pairwise comparison matrices are provided only for domains with significant overall differences (Clarity, Distractor quality, and Mean score). Bold *p*-values indicate statistical significance (*p* < 0.05). LLM, large language model; ICC, intraclass correlation coefficient; CI, confidence interval; Q1, first quartile; Q3, third quartile; NA, not applicable.

Overall comparisons showed no statistically significant differences among the five models in content rigor, educational usefulness, cognitive-level alignment, or overall usability (all *p* > 0.05), whereas statistically significant differences were observed for clarity, distractor quality, and mean score (all *p* < 0.001)(Table 3).

**Figure 4:**
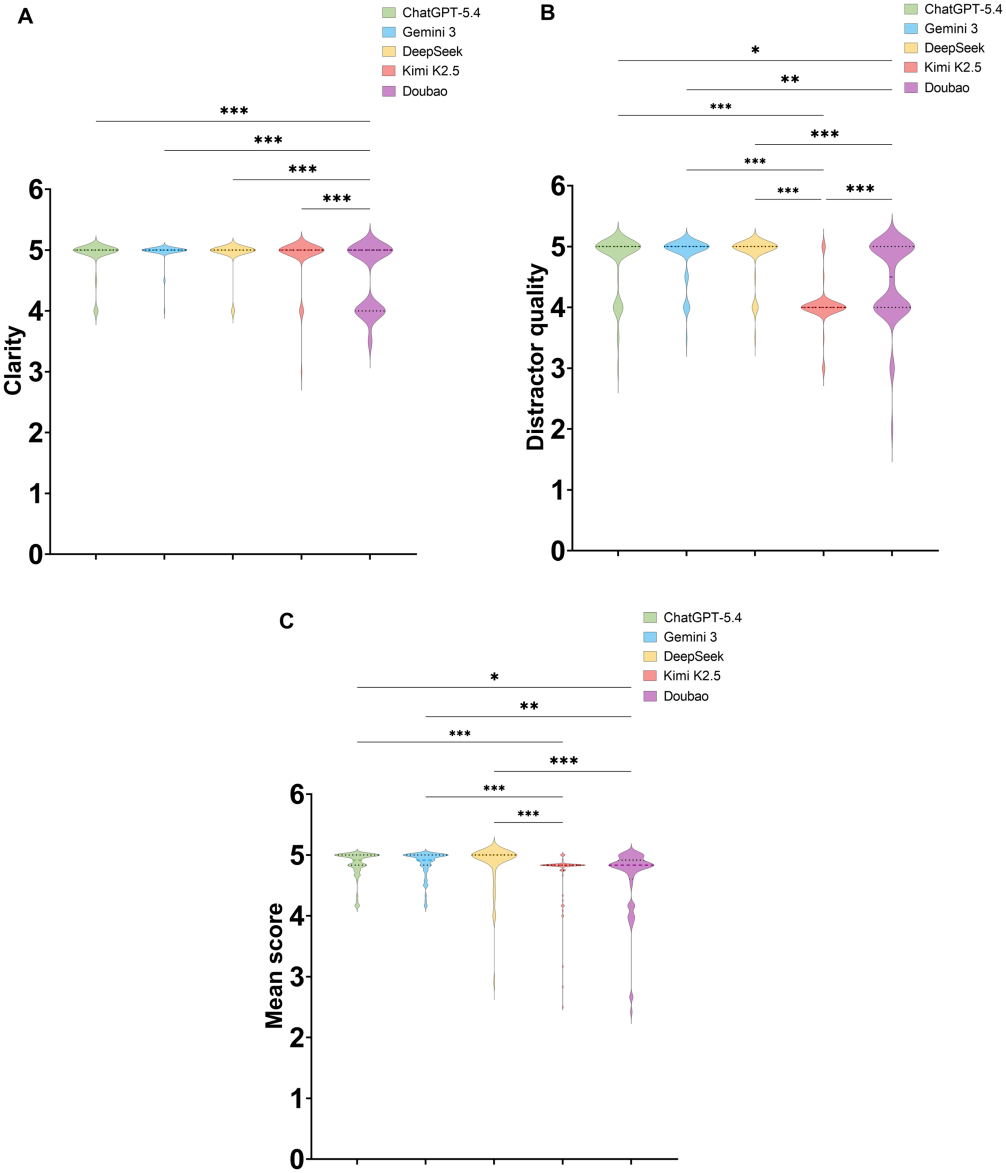
Comparison of expert subjective ratings across the five large language models (LLMs) for domains with significant overall between-model differences. **(A)** Clarity. **(B)** Distractor quality. **(C)** Mean score across the six subjective rating domains. Pairwise significant differences are indicated above the plots. *p < 0.05, **p < 0.01, ***p < 0.001.

For clarity, the median score was 5.00 (5.00, 5.00) for ChatGPT-5.4, Gemini 3, DeepSeek, and Kimi K2.5, whereas Doubao scored 5.00 (4.00, 5.00). *Post hoc* comparisons showed that Doubao scored significantly lower than the other four models, whereas no statistically significant differences were observed among those four models (Figure 4A).

For distractor quality, the median score was 5.00 (5.00, 5.00) for ChatGPT-5.4, Gemini 3, and DeepSeek; 4.50 (4.00, 5.00) for Doubao; and the lowest, 4.00 (4.00, 4.00), for Kimi K2.5. Post hoc comparisons showed that both Kimi K2.5 and Doubao scored significantly lower than ChatGPT-5.4, Gemini 3, and DeepSeek, and that Doubao scored significantly higher than Kimi K2.5 (Figure 4B).

For the mean score across the six subjective rating domains, DeepSeek ranked highest at 5.00 (5.00, 5.00), followed by ChatGPT-5.4 and Gemini 3, both at 4.92 (4.83, 5.00). Kimi K2.5 and Doubao showed relatively lower scores, at 4.83 (4.75, 4.83) and 4.83 (4.60, 4.92), respectively. Post hoc comparisons showed no statistically significant differences among ChatGPT-5.4, Gemini 3, and DeepSeek, but all three scored significantly higher than Kimi K2.5 and Doubao; no statistically significant difference was observed between Kimi K2.5 and Doubao (Figure 4C).

### 3.4 Supplementary classification results for overall usability

The supplementary classification results for overall usability are presented in Table 4. ChatGPT-5.4 showed the highest proportion of items classified as directly usable, at 55/60 (91.7%), followed by Gemini 3 at 54/60 (90.0%), DeepSeek at 52/60 (86.7%), Kimi K2.5 at 49/60 (81.7%), and Doubao at 47/60 (78.3%). In the category of usable after minor revision, the corresponding proportions were 8.3, 10.0, 11.7, 13.3, and 16.7%, respectively. No items were classified as usable after major revision for ChatGPT-5.4 or Gemini 3, whereas the corresponding proportions were 1.7% for DeepSeek, 5.0% for Kimi K2.5, and 5.0% for Doubao. Overall, although the Likert ratings for overall usability did not differ significantly among the five models, the supplementary classification results based on the pre-specified rules suggested that items generated by ChatGPT-5.4, Gemini 3, and DeepSeek showed relatively higher direct usability.

**Table 4:** Supplementary classification of the overall usability of HM MCQs generated by five LLMs.

Overall usability category	ChatGPT-5.4 (*n* = 60)	Gemini 3 (*n* = 60)	DeepSeek (*n* = 60)	Kimi K2.5 (*n* = 60)	Doubao (*n* = 60)
Directly usable, *n* (%)	55 (91.7%)	54 (90.0%)	52 (86.7%)	49 (81.7%)	47 (78.3%)
Usable after minor revision, *n* (%)	5 (8.3%)	6 (10.0%)	7 (11.7%)	8 (13.3%)	10 (16.7%)
Usable after major revision, *n* (%)	0 (0.0%)	0 (0.0%)	1 (1.7%)	3 (5.0%)	3 (5.0%)

This table provides a supplementary presentation of the overall usability domain from expert subjective ratings. Based on pre-specified decision rules, each generated question was further classified as directly usable, usable after minor revision, or usable after major revision before use. Data are presented as *n* (%), with percentages calculated using 60 generated questions per model as the denominator. HM, high myopia; LLM, large language model; MCQ, multiple-choice question.

### 3.5 Correlation analysis among textual output characteristics, generation efficiency, and expert ratings

Spearman correlation analysis was further performed to explore the relationships among expert subjective ratings, textual output characteristics, and generation efficiency. Overall, the subjective rating domains were predominantly positively correlated with one another, suggesting that high-quality items tended to perform well across multiple educational dimensions simultaneously. Among these, content rigor showed the strongest correlation with overall usability (*ρ* = 0.85, *p* < 0.05), and was also strongly positively correlated with educational usefulness (*ρ* = 0.74, *p* < 0.05) and mean score (*ρ* = 0.65, *p* < 0.05). Distractor quality also showed a strong positive correlation with mean score (*ρ* = 0.73, *p* < 0.05), and educational usefulness was strongly positively correlated with overall usability (*ρ* = 0.71, *p* < 0.05). In addition, mean score was significantly positively correlated with all subjective rating domains, with relatively stronger correlations observed for distractor quality (*ρ* = 0.73, *p* < 0.05), content rigor (*ρ* = 0.65, p < 0.05), overall usability (*ρ* = 0.64, *p* < 0.05), and educational usefulness (*ρ* = 0.63, *p* < 0.05).

Among the textual output characteristics, explanation length showed the strongest positive correlation with total response length (*ρ* = 0.90, *p* < 0.05). Total response length was also positively correlated with mean option length (*ρ* = 0.60, *p* < 0.05), response time (*ρ* = 0.57, *p* < 0.05), and stem length (*ρ* = 0.49, *p* < 0.05). Mean option length was positively correlated with option length SD (ρ = 0.47, p < 0.05), suggesting that longer options tended to be accompanied by greater imbalance in option length. Stem length was also positively correlated with explanation length (*ρ* = 0.34, *p* < 0.05) and response time (*ρ* = 0.28, *p* < 0.05).

With respect to the relationships between subjective ratings and textual features, longer textual outputs did not yield higher expert ratings. Specifically, clarity was negatively correlated with explanation length (*ρ* = −0.26, *p* < 0.05) and total response length (*ρ* = −0.24, *p* < 0.05). Distractor quality was negatively correlated with explanation length (*ρ* = −0.43, *p* < 0.05), total response length (*ρ* = −0.37, *p* < 0.05), and response time (*ρ* = −0.28, *p* < 0.05). Mean score was also negatively correlated with explanation length (*ρ* = −0.29, *p* < 0.05) and total response length (*ρ* = −0.24, *p* < 0.05). In addition, content rigor showed weak negative correlations with stem length (*ρ* = −0.14, *p* < 0.05), explanation length (*ρ* = −0.12, *p* < 0.05), and total response length (*ρ* = −0.15, *p* < 0.05), while overall usability likewise showed weak negative correlations with stem length (*ρ* = −0.15, *p* < 0.05), explanation length (*ρ* = −0.16, *p* < 0.05), and total response length (*ρ* = −0.18, *p* < 0.05).

**Figure 5:**
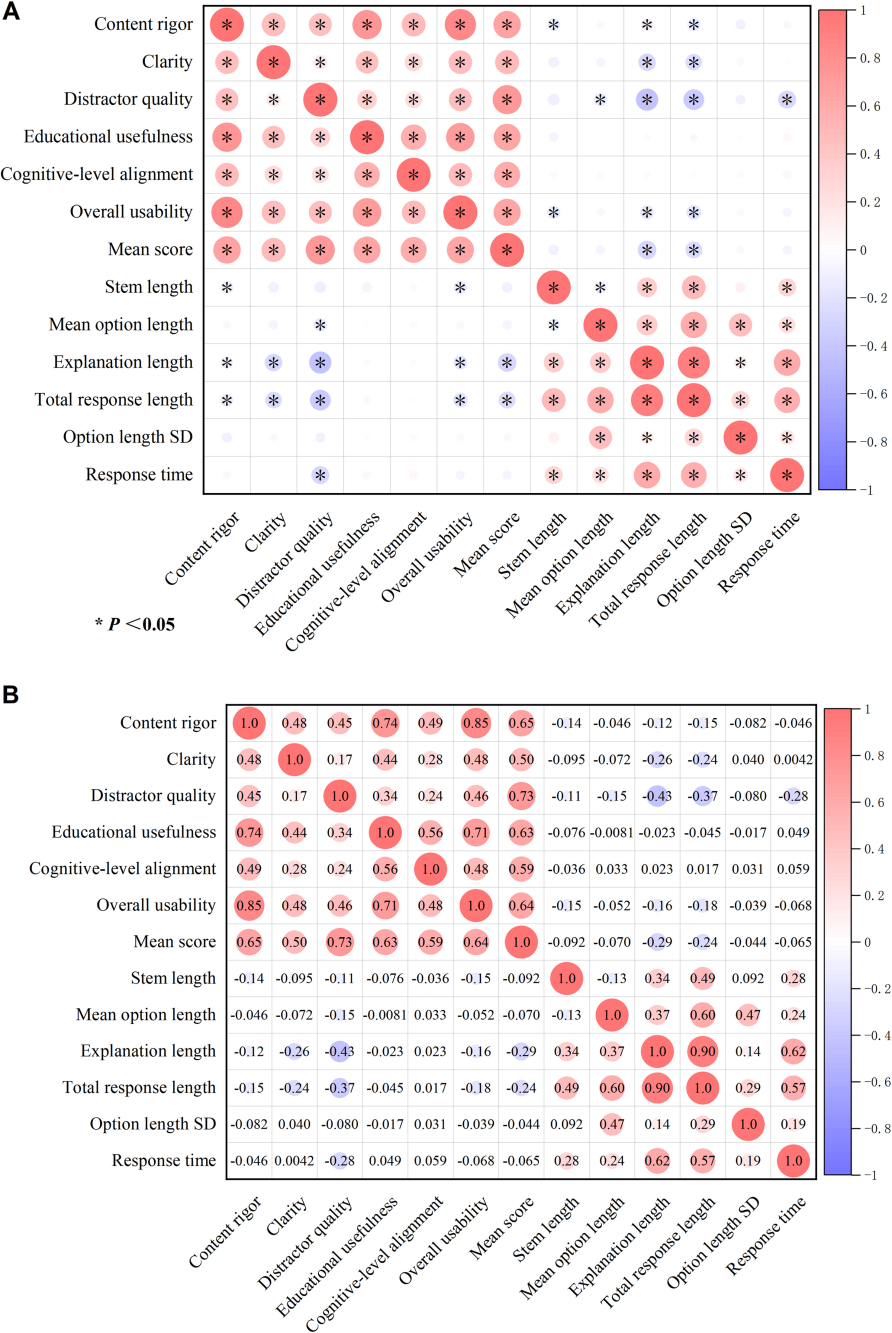
Correlation patterns among expert ratings, textual output characteristics, and generation efficiency. **(A)** Significance matrix of Spearman correlations among subjective rating domains, textual output characteristics, and response time; asterisks indicate statistically significant correlations (*p* < 0.05). **(B)** Spearman correlation coefficient matrix for the same variables; red indicates positive correlations and blue indicates negative correlations, with circle size reflecting the magnitude of the correlation coefficient. Standard deviation is abbreviated as SD.

Regarding generation efficiency, response time was positively correlated with explanation length (*ρ* = 0.62, *p* < 0.05), total response length (*ρ* = 0.57, *p* < 0.05), stem length (*ρ* = 0.28, *p* < 0.05), mean option length (*ρ* = 0.24, *p* < 0.05), and option length SD (*ρ* = 0.19, *p* < 0.05), indicating that outputs requiring longer response times were generally accompanied by longer textual content. However, response time did not show an advantage consistent with higher subjective quality; instead, it was negatively correlated with distractor quality (*ρ* = −0.28, *p* < 0.05), suggesting that under the conditions of this study, slower generation speed did not imply better educational quality (Figure 5).

## 4 Discussion

This study systematically compared the performance of five publicly accessible LLMs in generating HM-related single-best-answer MCQs for educational use. Overall, all models were able to stably generate items with structural completeness and format compliance under a standardized Chinese prompt and a single-round generation workflow, suggesting that current mainstream LLMs have already acquired a relatively strong basic capacity for standardized item generation. However, certain differences were observed among models in correct answer accuracy, textual output style, clarity, distractor quality, and mean subjective score. Overall, ChatGPT-5.4 and Gemini 3 performed best in terms of answer accuracy; DeepSeek showed the highest descriptive performance in mean subjective score, although the difference was not statistically significant compared with ChatGPT-5.4 and Gemini 3; and Kimi K2.5 and Doubao still appeared to have room for further improvement in some dimensions of educational quality. In addition, based on the supplementary classification results, ChatGPT-5.4, Gemini 3, and DeepSeek showed relatively more favorable descriptive trends in direct usability. These findings suggest that, in the disease-specific educational scenario of HM, the major challenge for LLMs is no longer whether they can generate an item as instructed, but whether they can consistently generate items that are medically accurate, educationally high-quality, and directly usable.

The selection of HM as the evaluation scenario has clear relevance to eye health and public health. The burden of myopia and HM continues to rise, and HM is not merely a more severe refractive state, but rather an important eye health condition closely associated with posterior scleral ectasia, macular lesions, retinal detachment, choroidal neovascularization, and the risk of long-term visual impairment (35, 36). Accordingly, HM-related teaching inherently spans multiple dimensions, including basic concepts, image interpretation, complication identification, risk stratification, follow-up, and management decision-making, and therefore provides a more comprehensive test of model capability in disease-specific educational content generation than isolated single-point knowledge tasks. Compared with previous studies that have focused more on patient question-answering or the quality of myopia care recommendations (37–39), recent evidence has also suggested that multimodal large language models may have potential in ophthalmic image-based case interpretation, including both multiple-choice and open-ended response tasks (40), further underscoring the expanding role of LLMs in ophthalmic education and assessment. Against this broader background, the present study further extends the evaluation scenario to standardized item generation for specialty training, making its topic selection more closely aligned with the practical needs of eye health workforce development and digital educational resource construction (41).

One of the most direct findings of this study was that all five models achieved 100.0% in structurally qualified output rate, final structurally complete output rate, and format compliance rate. This indicates that, under conditions in which task boundaries are clear, the output template is explicit, and prompt constraints are sufficiently specified, current LLMs are already able to complete the task of template-based item generation with relatively high stability. Taken together with previous systematic reviews and empirical studies on LLM-generated medical examination items, LLMs do appear to have strong potential for MCQ generation, but their outputs are not inherently equivalent to high-quality test items that can be directly used in formal assessment. Existing studies have shown, on the one hand, that such models can generate basically usable items with relatively high efficiency; on the other hand, they have repeatedly identified persistent problems, including factual errors, insufficient relevance, poor difficulty calibration, and suboptimal cognitive-level targeting, and many generated items still require further revision or expert review before use (42). In other words, in the context of educational content generation, structural adherence is gradually shifting from a major bottleneck to a relatively attainable prerequisite; the factors that truly differentiate models remain medical factual accuracy, cognitive-level alignment, item relevance, and fine-grained educational quality control. This interpretation is broadly consistent with current review evidence in medicine and medical education. LLMs are widely considered to have substantial potential in personalized learning support, case simulation, patient education material generation, medical information translation, and partial automation of teaching workflows. At the same time, however, content accuracy, output stability, ethical risks, and reliability in real-world application settings remain major issues limiting their adoption in formal teaching and other high-stakes applications (43, 44). More broadly, regulatory and applied research has pointed out that LLMs for medical use still face core obstacles such as high output variability, limited intrinsic interpretability, and the risk of hallucinations. Moreover, in real diagnostic reasoning experiments, merely granting physicians access to LLMs has not significantly improved their diagnostic reasoning performance. This further suggests that strong performance in benchmarking tasks or template-based generation does not automatically translate into equally stable and trustworthy value in real educational or clinical settings (45).

Differences in textual output style across models also warrant attention. ChatGPT-5.4 tended to generate relatively shorter stems and responded more quickly, whereas DeepSeek and Kimi K2.5 tended to produce longer stems; Kimi K2.5 and Doubao, in turn, generated longer explanations and longer total responses. It should be emphasized that longer text does not inherently indicate higher educational quality. Our results showed that, although Kimi K2.5 and Doubao produced longer explanations, their mean subjective scores were not correspondingly superior. By contrast, DeepSeek achieved the highest mean subjective score while maintaining relatively high answer accuracy, suggesting that the relationship between information quantity and educational effectiveness is not simply linear. One reasonable inference is that better items do not depend merely on greater text volume, but rather on a balance among information organization, focused wording, and explanatory effectiveness. Therefore, in the context of HM educational item generation, the ideal output is more likely to be an item that is sufficiently explanatory without being excessively verbose, rather than the longest possible item.

Distractor quality was one of the most educationally meaningful discriminatory indicators in this study. Unlike content rigor, educational usefulness, and cognitive-level alignment, which were generally high across models, distractor quality showed more apparent between-model differences, with Kimi K2.5 and Doubao performing relatively less well. One possible explanation for the negative association between distractor quality and explanation length is that models producing more verbose outputs may distribute attention across peripheral details rather than prioritizing the key discriminatory clinical cue. In MCQ writing, high-quality distractors are typically concise, plausible, and closely linked to common diagnostic or management confusions. When explanations become unnecessarily long, the same generative tendency may also produce distractors that are overly elaborate, partially correct, or clinically tangential, thereby reducing their precision and educational usefulness. This domain may be more discriminatory because, for MCQs, the most difficult part to automate is often neither writing a stem nor providing a correct answer, but rather designing incorrect options that are both plausibly misleading and not effectively equivalent to the correct answer under clinical boundary conditions. Previous research in educational measurement has shown that item-writing flaws and poor distractors can directly affect item discrimination, examination validity, and even judgments of learner performance (46, 47). In a disease-specific topic such as HM, which involves disease definition, pathological changes, complication management, and follow-up strategies, distractors that are overly simple, semantically repetitive, or only partially incorrect may weaken item quality and mislead learners’ knowledge construction. Therefore, the quality of distractor design may reflect the real usability of LLMs in specialist educational settings more effectively than whether an item merely appears superficially well formed.

From an application perspective, the findings of this study support positioning LLMs as assistive tools for HM educational resource development rather than as fully autonomous item-generation systems that replace experts. Current systematic reviews in medical education suggest that LLMs have considerable potential for educational content generation, learning support, and expansion of assessment resources, but that rigorous validation and oversight are still required before they can be introduced into high-stakes educational settings (48–50). In ophthalmology, existing systematic reviews likewise indicate that research on LLMs is growing rapidly overall, but that real-world deployment, non-English settings, disease-specific training tasks, and standardized evaluation workflows remain relatively underexplored (51). Under standardized Chinese prompts and real-world public web-based conditions, the present study showed that LLMs can efficiently generate draft items for the HM educational scenario, with ChatGPT-5.4, Gemini 3, and DeepSeek in particular showing substantial potential in terms of correctness and direct usability. This implies that, in settings such as residency training, continuing education, primary eye health training, and frequently updated item banks, LLMs may be useful for draft generation, expansion of teaching materials, and improvement of item-development efficiency (52). Nevertheless, a more feasible practical pathway remains a human–AI collaborative workflow of “model drafting–expert review–standardized inclusion in the item bank,” rather than direct use in formal assessment without prior review (53, 54).

This study also has several strengths. First, it focused on HM as a disease-specific scenario with substantial eye health burden, long-term management needs, and educational representativeness, rather than broadly addressing general ophthalmic item generation, thereby enhancing both the focus and practical relevance of the research question. Second, the use of a unified item-generation blueprint, a standardized Chinese prompt template, uniform web-based access conditions, and a single-round generation workflow improved the fairness of cross-model comparisons to a considerable extent. Third, by incorporating objective evaluation, expert subjective ratings, inter-rater agreement analysis, and supplementary usability classification, this study was able to provide a relatively comprehensive picture of model performance in disease-specific educational item generation.

Several limitations should also be acknowledged. First, this study focused exclusively on HM, and therefore the findings may not be directly generalizable to other ophthalmic subspecialties, such as diabetic retinopathy, glaucoma, cataract, or neuro-ophthalmology. Second, all tests were conducted through public web interfaces, and underlying hidden parameters, platform-level system prompts, and model version updates could not be fully controlled; accordingly, the results are subject to a certain degree of platform dependence and temporal sensitivity. In particular, the recorded response time reflected end-to-end user-visible generation under web-based conditions rather than pure model inference speed, and may have been influenced by server load, network latency, and platform traffic. Third, although this study included blinded ratings by two experts and demonstrated good agreement, subjective evaluation cannot be entirely separated from expert judgment. In addition, some models showed relatively recognizable textual styles, which may have introduced a limited risk of partial unblinding during scoring. Most items in this study also received relatively high scores on some subjective rating domains, suggesting a possible ceiling effect that may have limited the scale’s ability to discriminate subtle between-model differences. In addition, the pragmatic sample size used in this benchmarking study may have reduced statistical power for detecting small between-model differences. Future studies may consider higher-resolution rating systems, a larger number of raters, or additional distribution-based indicators to improve evaluative sensitivity. Fourth, this study assessed generation quality rather than real examination performance, and did not include response data from residents or students; moreover, no human-generated control set was included as an external benchmark. Therefore, the actual difficulty, discrimination, and psychometric properties of the generated items still require further validation. Fifth, a single-round generation design was adopted to ensure model comparability, but this may also have underestimated the potential performance of some models under multi-turn interactive optimization. Future studies could extend this framework to other disease-specific ophthalmic topics and integrate real learner responses, psychometric validation, human-written reference items, and comparisons across different prompting strategies, different languages, and even multimodal inputs, in order to build a more mature human–AI collaborative framework for eye health educational content generation (55). In addition, the correlation analyses in this study were primarily exploratory and descriptive. More complex modeling that accounts for multiple comparisons and model-level clustering effects was not performed; therefore, these correlation findings are more appropriate for hypothesis generation than for strong inference.

## 5 Conclusion

Overall, this study suggests that LLMs have already demonstrated substantial potential for structured item generation in the context of HM-related single-best-answer MCQ development for educational use. However, their high-quality application still depends on the control of factual accuracy, optimization of distractors, and the incorporation of expert review mechanisms. At the current stage, a more feasible pathway is not fully automated item generation, but rather the integration of LLMs into a human–AI collaborative generation framework for eye health education and training under a standardized evaluation framework.

## Data Availability

The original contributions presented in the study are included in the article/Supplementary material, further inquiries can be directed to the corresponding authors.
