# Supplementary material for: Benchmarking publicly accessible large language models for high-myopia multiple-choice question generation in digital ophthalmic education and public health training
Source: Front Public Health. 2026 May 5;14:1843045. doi: 10.3389/fpubh.2026.1843045 (PMC13183845; doi:10.3389/fpubh.2026.1843045)
Supplement: Supplementary file 1 [file Supplementary_file_1.DOCX]

**Table S1. Pairwise Comparison Matrix for Stem Length Across the Five LLMs**

| **Z-values \ P-values** | **ChatGPT-5.4** | **Gemini 3** | **DeepSeek** | **Kimi K2.5** | **Doubao** |
| --- | --- | --- | --- | --- | --- |
| **ChatGPT-5.4** | — | 0.041 | 0.000 | 0.000 | 0.000 |
| **Gemini 3** | 2.868 | — | 0.027 | 0.048 | 1.000 |
| **DeepSeek** | 5.864 | 2.997 | — | 1.000 | 1.000 |
| **Kimi K2.5** | 5.685 | 2.817 | 0.179 | — | 1.000 |
| **Doubao** | 4.248 | 1.381 | 1.616 | 1.436 | — |

***Note:*** The lower triangle presents absolute |Z| values (standardized test statistics) from post hoc pairwise comparisons following the Kruskal–Wallis test, whereas the upper triangle presents Bonferroni-adjusted two-sided P-values. Values shown as 0.000 indicate P < 0.001. Diagonal dashes indicate within-model comparisons were not applicable.

**Table S2. Pairwise Comparison Matrix for Mean Option Length Across the Five LLMs**

| **Z-values \ P-values** | **ChatGPT-5.4** | **Gemini 3** | **DeepSeek** | **Kimi K2.5** | **Doubao** |
| --- | --- | --- | --- | --- | --- |
| **ChatGPT-5.4** | — | 0.318 | 1.000 | 0.083 | 0.003 |
| **Gemini 3** | 2.147 | — | 0.004 | 0.000 | 0.000 |
| **DeepSeek** | 1.369 | 3.516 | — | 1.000 | 0.235 |
| **Kimi K2.5** | 2.640 | 4.786 | 1.270 | — | 1.000 |
| **Doubao** | 3.634 | 5.781 | 2.265 | 0.995 | — |

***Note:*** The lower triangle presents absolute |Z| values (standardized test statistics) from post hoc pairwise comparisons following the Kruskal–Wallis test, whereas the upper triangle presents Bonferroni-adjusted two-sided P-values. Values shown as 0.000 indicate P < 0.001. Diagonal dashes indicate within-model comparisons were not applicable.

**Table S3. Pairwise Comparison Matrix for Explanation Length Across the Five LLMs**

| **Z-values \ P-values** | **ChatGPT-5.4** | **Gemini 3** | **DeepSeek** | **Kimi K2.5** | **Doubao** |
| --- | --- | --- | --- | --- | --- |
| **ChatGPT-5.4** | — | 0.009 | 0.000 | 0.000 | 0.000 |
| **Gemini 3** | 3.312 | — | 0.000 | 0.000 | 0.000 |
| **DeepSeek** | 4.117 | 7.429 | — | 0.000 | 0.000 |
| **Kimi K2.5** | 9.466 | 12.778 | 5.349 | — | 1.000 |
| **Doubao** | 9.313 | 12.625 | 5.195 | 0.153 | — |

***Note:***The lower triangle presents absolute |Z| values (standardized test statistics) from post hoc pairwise comparisons following the Kruskal–Wallis test, whereas the upper triangle presents Bonferroni-adjusted two-sided P-values. Values shown as 0.000 indicate P < 0.001. Diagonal dashes indicate within-model comparisons were not applicable.

**Table S4. Pairwise Comparison Matrix for Total Response Length Across the Five LLMs**

| **Z-values \ P-values** | **ChatGPT-5.4** | **Gemini 3** | **DeepSeek** | **Kimi K2.5** | **Doubao** |
| --- | --- | --- | --- | --- | --- |
| **ChatGPT-5.4** | — | 0.391 | 0.000 | 0.000 | 0.000 |
| **Gemini 3** | 2.063 | — | 0.000 | 0.000 | 0.000 |
| **DeepSeek** | 4.945 | 7.008 | — | 0.001 | 0.001 |
| **Kimi K2.5** | 8.935 | 10.999 | 3.991 | — | 1.000 |
| **Doubao** | 8.807 | 10.871 | 3.863 | 0.128 | — |

***Note:*** The lower triangle presents absolute |Z| values (standardized test statistics) from post hoc pairwise comparisons following the Kruskal–Wallis test, whereas the upper triangle presents Bonferroni-adjusted two-sided P-values. Values shown as 0.000 indicate P < 0.001. Diagonal dashes indicate within-model comparisons were not applicable.

**Table S5. Pairwise Comparison Matrix for Option Length SD Across the Five LLMs**

| **Z-values \ P-values** | **ChatGPT-5.4** | **Gemini 3** | **DeepSeek** | **Kimi K2.5** | **Doubao** |
| --- | --- | --- | --- | --- | --- |
| **ChatGPT-5.4** | — | 0.863 | 0.004 | 0.003 | 1.000 |
| **Gemini 3** | 1.715 | — | 0.669 | 0.541 | 1.000 |
| **DeepSeek** | 3.547 | 1.832 | — | 1.000 | 0.031 |
| **Kimi K2.5** | 3.641 | 1.926 | 0.094 | — | 0.023 |
| **Doubao** | 0.591 | 1.124 | 2.957 | 3.050 | — |

***Note:*** The lower triangle presents absolute |Z| values (standardized test statistics) from post hoc pairwise comparisons following the Kruskal–Wallis test, whereas the upper triangle presents Bonferroni-adjusted two-sided P-values. Values shown as 0.000 indicate P < 0.001. Diagonal dashes indicate within-model comparisons were not applicable.

**Table S6. Pairwise Comparison Matrix for Response Time Across the Five LLMs**

| **Mean difference \ P-values** | **ChatGPT-5.4** | **Gemini 3** | **DeepSeek** | **Kimi K2.5** | **Doubao** |
| --- | --- | --- | --- | --- | --- |
| **ChatGPT-5.4** | — | 0.000 | 0.000 | 0.000 | 0.000 |
| **Gemini 3** | 2.456 | — | 0.000 | 0.000 | 0.000 |
| **DeepSeek** | 7.356 | 9.812 | — | 0.876 | 0.000 |
| **Kimi K2.5** | 6.852 | 9.308 | 0.504 | — | 0.000 |
| **Doubao** | 1.860 | 4.316 | 5.497 | 4.993 | — |

***Note:*** The lower triangle presents absolute mean differences (in seconds) from Games-Howell post hoc pairwise comparisons following Welch's ANOVA, whereas the upper triangle presents Games-Howell-adjusted two-sided P-values. Values shown as 0.000 indicate P < 0.001. Diagonal dashes indicate within-model comparisons were not applicable.

**Table S7. Pairwise Comparison Matrix for Clarity Across the Five LLMs**

| **\|Z\| values \ P-values** | **ChatGPT-5.4** | **Gemini 3** | **DeepSeek** | **Kimi K2.5** | **Doubao** |
| --- | --- | --- | --- | --- | --- |
| **ChatGPT-5.4** | — | 1.000 | 1.000 | 1.000 | 0.000 |
| **Gemini 3** | 1.010 | — | 1.000 | 1.000 | 0.000 |
| **DeepSeek** | 0.687 | 0.323 | — | 1.000 | 0.000 |
| **Kimi K2.5** | 0.144 | 0.866 | 0.543 | — | 0.000 |
| **Doubao** | 4.970 | 5.980 | 5.657 | 5.114 | — |

***Note:*** The lower triangle presents absolute |Z| values (standardized test statistics) from post hoc pairwise comparisons following the Kruskal–Wallis test, whereas the upper triangle presents Bonferroni-adjusted two-sided P-values. Values shown as 0.000 indicate P < 0.001. Diagonal dashes indicate within-model comparisons were not applicable.

**Table S8. Pairwise Comparison Matrix for Distractor Quality Across the Five LLMs**

| **\|Z\| values \ P-values** | **ChatGPT-5.4** | **Gemini 3** | **DeepSeek** | **Kimi K2.5** | **Doubao** |
| --- | --- | --- | --- | --- | --- |
| **ChatGPT-5.4** | — | 1.000 | 1.000 | 0.000 | 0.026 |
| **Gemini 3** | 0.691 | — | 1.000 | 0.000 | 0.002 |
| **DeepSeek** | 1.032 | 0.342 | — | 0.000 | 0.001 |
| **Kimi K2.5** | 7.630 | 8.321 | 8.663 | — | 0.000 |
| **Doubao** | 3.015 | 3.706 | 4.047 | 4.616 | — |

***Note:*** The lower triangle presents absolute |Z| values (standardized test statistics) from post hoc pairwise comparisons following the Kruskal–Wallis test, whereas the upper triangle presents Bonferroni-adjusted two-sided P-values. Values shown as 0.000 indicate P < 0.001. Diagonal dashes indicate within-model comparisons were not applicable.

**Table S9. Pairwise Comparison Matrix for Mean Score Across the Five LLMs**

| **\|Z\| values \ P-values** | **ChatGPT-5.4** | **Gemini 3** | **DeepSeek** | **Kimi K2.5** | **Doubao** |
| --- | --- | --- | --- | --- | --- |
| **ChatGPT-5.4** | — | 1.000 | 0.248 | 0.000 | 0.013 |
| **Gemini 3** | 0.384 | — | 0.627 | 0.000 | 0.003 |
| **DeepSeek** | 2.245 | 1.862 | — | 0.000 | 0.000 |
| **Kimi K2.5** | 4.103 | 4.487 | 6.349 | — | 1.000 |
| **Doubao** | 3.225 | 3.608 | 5.470 | 0.879 | — |

***Note:*** The lower triangle presents absolute |Z| values (standardized test statistics) from post hoc pairwise comparisons following the Kruskal–Wallis test, whereas the upper triangle presents Bonferroni-adjusted two-sided P-values. Values shown as 0.000 indicate P < 0.001. Diagonal dashes indicate within-model comparisons were not applicable.
